# Long noncoding RNA LCAT1 functions as a ceRNA to regulate RAC1 function by sponging miR-4715-5p in lung cancer

**DOI:** 10.1186/s12943-019-1107-y

**Published:** 2019-11-29

**Authors:** Juze Yang, Qiongzi Qiu, Xinyi Qian, Jiani Yi, Yiling Jiao, Mengqian Yu, Xufan Li, Jia Li, Chunyi Mi, Jisong Zhang, Bingjian Lu, Enguo Chen, Pengyuan Liu, Yan Lu

**Affiliations:** 1 Department of Respiratory Medicine, Sir Run Run Shaw Hospital and Institute of Translational Medicine, Zhejiang University School of Medicine, Zhejiang, 310016 Hangzhou China; 2 Center for Uterine Cancer Diagnosis & Therapy Research of Zhejiang Province, Women’s Reproductive Health Key Laboratory of Zhejiang Province, Department of Gynecologic Oncology, Women’s Hospital and Institute of Translational Medicine, Zhejiang University School of Medicine, Zhejiang, 310006 Hangzhou China; 3 Center of Systems Molecular Medicine, Department of Physiology, Medical College of Wisconsin, Milwaukee, WI 53226 USA

**Keywords:** Long noncoding RNAs, Lung cancer, miR-4715-5p, Oncogene, RAC1

## Abstract

**Introduction:**

Long noncoding RNAs (lncRNAs) are emerging as key players in the development and progression of cancer. However, the biological role and clinical significance of most lncRNAs in lung carcinogenesis remain unclear. In this study, we identified and explored the role of a novel lncRNA, lung cancer associated transcript 1 (LCAT1), in lung cancer.

**Methods:**

We predicted and validated LCAT1 from RNA-sequencing (RNA-seq) data of lung cancer tissues. The LCAT1–miR-4715-5p–RAC1 axis was assessed by dual-luciferase reporter and RNA immunoprecipitation (RIP) assays. Signaling pathways altered by LCAT1 knockdown were identified using RNA-seq. Furthermore, the mechanism of LCAT1 was investigated using loss-of-function and gain-of-function assays in vivo and in vitro.

**Results:**

LCAT1 is an oncogene that is significantly upregulated in lung cancer tissues and associated with poor prognosis. LCAT1 knockdown caused growth arrest and cell invasion in lung cancer cells in vitro, and inhibited tumorigenesis and metastasis in the mouse xenografts. Mechanistically, LCAT1 functions as a competing endogenous RNA for miR-4715-5p, thereby leading to the upregulation of the activity of its endogenous target, Rac family small GTPase 1 (RAC1). Moreover, EHop-016, a small molecule inhibitor of RAC1, as an adjuvant could improve the Taxol monotherapy against lung cancer cells in vitro.

**Conclusions:**

LCAT1–miR-4715-5p–RAC1/PAK1 axis plays an important role in the progression of lung cancer. Our findings may provide valuable drug targets for treating lung cancer. The novel combination therapy of Taxol and EHop-016 for lung cancer warrants further investigation, especially in lung cancer patients with high LCAT1 expression.

## Introduction

Lung cancer is the most commonly diagnosed cancer and the leading cause of cancer death globally [1]. The overall 5-year survival rate of patients with lung cancer is approximately 15%, which has remained unchanged in the last several decades despite advances in surgical techniques and molecular targeting therapy [2]. Poor outcomes of lung cancer are associated with diagnosis at advanced stages and the propensity for metastasis. Currently, there are no effective biomarkers for early diagnosis of lung cancer. There is a limited understanding of the molecular pathogenesis of lung cancer [3, 4]. Therefore, there is an urgent need to identify new biomarkers for early diagnosis and prognosis and to identify new drug targets combating the proliferation and metastasis of lung cancer cells.

Non-coding RNAs (ncRNAs) compose the large majority (approximately 98%) of the human transcriptome [5, 6]. Long non-coding RNAs (lncRNAs) are a large and diverse class of ncRNAs whose transcripts are longer than 200 nucleotides with limited or no protein-coding capacity [6–8]. Numerous studies have reported that lncRNAs participate in diverse biological functions such as cell proliferation, stem cell differentiation, immune response, and disease pathogenesis [9–11]. The molecular mechanisms by which lncRNAs exert their biological function are diverse and complex [12–14]. For example, H19 expression modulates chromatin and nucleosome assembly, resulting in gene imprinting [15, 16]. In addition, LINC00673 promotes cell proliferation and progression through sponging miR-150-5p [17]. Notably, lncRNAs play a vital role in human carcinogenesis. However, the molecular mechanisms underlying the role of lncRNAs in carcinogenesis require further investigation.

In the present study, we identified a novel lncRNA, lung cancer associated transcript 1 (LCAT1). The biological function and molecular mechanism of LCAT1 are unexplored. LCAT1 is markedly upregulated in lung cancer tissues and is associated with poor prognosis. Functional assays demonstrated that LCAT1 promotes lung cancer cell proliferation and progression in vitro and in vivo. Furthermore, mechanistic analysis reveals that LCAT1 functions as a competing endogenous RNA (ceRNA) to regulate the expression and function of Rac family small GTPase 1 (RAC1) through competitively binding with miR-4715-5p. Taken together, LCAT1 is an oncogenic regulator of lung cancer development and progression and is a valuable biomarker and therapeutic target.

## Materials and methods

### Cell culture

Four human lung adenocarcinoma cell lines (A549, Calu1, H1299, and HOP62) were procured from the American Type Culture Collection (ATCC). The cell lines were maintained in RPMI-1640 medium (Gibco, Waltham, MA). All cell lines were supplemented with 10% fetal bovine serum (FBS; Gibco), 100 U/mL penicillin, and 100 mg/mL streptomycin and grown at 37 °C and 5% CO_2_.

### RNA extraction and RT-qPCR

RNA samples from frozen lung tissue specimens and cultured cells were extracted using Trizol Reagent (Invitrogen, Carlsbad, CA). Complementary DNA (cDNA) from 1 μg total RNA was synthesized using SuperScript II (Vazyme, Nanjing, China). The amplification reaction volume was 10 μL containing SYBR Green PCR Master Mix (Vazyme), 1 μL cDNA, and amplification primers. The actin mRNA was used for normalization. The relative expression of each examined gene was determined in technical triplicates.

### Cell transfection

LCAT1 and RAC1 short interfering RNA (siRNAs) and an hsa-miR-4715-5p inhibitor were synthesized by Shanghai Gene Pharma Co., Ltd. (Shanghai, China). An hsa-miR-4715-5p mimic was synthesized by Ribo Co., Ltd. (Guangdong, China) (Additional file 1: Table S1). SiRNAs were transfected at a final concentration of 50 nM using GeneMute™ reagent (SignaGen™ Laboratories, Rockville, MD), following the manufacturer’s instruction.

### Cell proliferation assay and colony formation

An equal number of cells were plated in 96-well plates using 5 wells for technical replicates. Cell viability was measured using a cell counting kit-8 (CCK8) kit (Dojindo Laboratories, Kumamoto, Japan). Absorbance was measured at a wavelength of 450 nm. Cell proliferation was also assessed using a Cell-Light EdU DNA cell proliferation kit (RiboBio, Guangzhou, China), following the manufacturer’s instructions. For the colony formation assay, lung cancer cells were seeded into 6-well plates with 5000 cells/well. The cells were cultured for 10 days before they were fixed in formalin and stained with crystal violet. The colonies were counted, and the results were reported as the relative colony number.

### Flow cytometry analysis of cell cycle

The cells were harvested and washed with phosphate buffer saline (PBS). The pellet was then resuspended, fixed in 70% prechilled methanol, and stored overnight at 4 °C. The cells were washed again with PBS followed by addition of 200 μL staining solution (0.1% [v/v] Triton X-100, 1 μg/mL DAPI in PBS). The final mixture was incubated for 30 min in the dark before flow cytometry analysis. The experiments were performed in triplicate and repeated 3 times.

### Invasion and migration assays

In vitro migration and invasion assays were performed using transwell chambers. Lung cancer cells were transfected with siRNA or negative control for 24 h. The cells were cultured with serum-free RPMI 1640 medium for 24 h, then detached and resuspended in serum-free RPMI 1640 medium. Cells were concentrated to 3 × 10^4^ cells in 300 uL cell suspension and then added to the upper chamber for the migration assay or the upper chamber coated with Matrigel for the invasion assay. The RPMI 1640 medium supplemented with 10% FBS was added to the bottom chamber. Cells that migrated or invaded into the bottom chamber were stained with 0.1% crystal violet. Images were captured from each membrane and the number of migratory cells was counted under a microscope.

### 5′ and 3′ RACE assay

A 5′ RACE assay and 3′ RACE assay were performed to determine the full length of LCAT1 using a SMARTer RACE cDNA Amplification kit (Clontech, Takara, Japan), following the manufacturer’s instructions.

### Subcellular fractionation

We harvested 2 × 10^7^ cells, washed them with ice-cold PBS, and then resuspended the cells in the ice-cold cytoplasmic lysis buffer (0.15% NP-40, 10 mM Tris pH 7.5, 150 mM NaCl) for 5 min on ice. The lysates were transferred into ice-cold sucrose buffer and centrifuged at 13,000 *g* for 10 min at 4 °C. The supernatant (~ 700 μL) was collected as the cytoplasmic fraction.

### Luciferase assay

The whole sequence of LCAT1 (or RAC1 3′ UTR) was inserted into the psiCHECK2 basic construct. 293 T cells were transfected with 0.5 μg reporter construct and 50 nM siRNA (or miRNA mimic) per well using Lipofectamine 3000 (Invitrogen, Cat# L3000–015). After 12 h of transfection, we replaced the transfection medium with complete culture medium. After 48 h culture, the cells were lysed with passive lysis buffer (Promega, Cat# E1910), and the reporter gene expression was assessed using a Dual Luciferase reporter assay system (Promega, Cat# E1910). All transfection assays were carried out in triplicate.

### Western blot

Cells were suspended in lysis buffer (50 mM Tris-HCl PH 8.0, 1% SDS, 1 mM EDTA, 5 mM DTT, 10 mM PMSF, 1 mM NaF, 1 mM Na_3_VO_4_, and protease inhibitor cocktail), and then denatured in boiling water for 10 min. The cellular lysates were centrifuged at 13,000 rpm for 30 min. The protein concentration was determined using a BCA assay (Thermo Fisher Scientific, Waltham, MA, USA). Equal amount of proteins (40 μg) was used to perform sodium dodecyl polyacrylamide gel electrophoresis (SDS-PAGE) using 10% gel. The proteins were then transferred onto a polyvinylidene fluoride (PVDF) membrane. The membrane was blocked with 5% skim milk and incubated with the antibodies. The antibodies used included rabbit anti-Wee1, anti-Cyclin B1, anti-Cyclin D1, anti-cyclin E1, anti-PAK1 and anti-RhoA, mouse anti-Rac1, anti-CDK6 and anti-Cyclin A2 (Additional file 1: Table S1). Immunoreactive bands were developed by enhanced chemiluminescence reaction (Pierce) following standard protocols.

### In vivo assay

Briefly, 5–6 week old female athymic nude mice (BALB/c Nude) were used for the xenograft model. A549 cells stably expressing shCtrl or shLCAT1 were dissociated using trypsin and washed twice with sterilized PBS. Then, 0.2 mL of PBS containing 3 × 10^6^ cells was subcutaneously inoculated into the flank of mice. Mice were monitored every 3 days for tumor growth, and the tumor size was measured using a caliper. Three weeks after inoculation, the mice were sacrificed adhering to the policy on the humane treatment of tumor-bearing animals. To further investigate the effect on tumor invasion in vivo, 2 × 10^6^ scramble or shLCAT1 cells were injected intravenously into the tail vein. Five minutes following injection, 1.5 mg luciferin (Gold Biotech, St Louis, MO, USA) was administered to monitor metastases using an IVIS@ Lumina II system (Caliper Life Sciences, Hopkinton, MA, USA). Two-sample t-test with two-tailed *P*-values was performed to detect the difference in tumor metastasis between the two groups. All experiments were performed in accordance with the Guide for the Care and Use of Laboratory Animals (NIH publication 80–23, revised 1996), with the approval of the Zhejiang University, Hangzhou, China.

### Library preparation for RNA sequencing

Transcriptome analysis of LCAT1 knockdown and scrambled control lung cancer cells was conducted using RNA sequencing (RNA-seq) as described previously [18]. Briefly, total RNA was isolated using TRIzol according to the manufacturer’s instructions (Invitrogen). cDNA libraries were prepared using a TruSeq RNA Sample Preparation Kit (Illumina). Libraries were quantified using qPCR according to the Illumina’s qPCR quantification guide to ensure uniform cluster density. Samples were multiplexed with 12 samples per lane and paired-end sequenced with an Illumina HiSeq X10 (Additional file 2: Table S2).

### Analysis of RNA-seq data

Transcriptome data were mapped with Tophat v2 using the spliced mapping algorithm [19]. A set of both known and novel transcripts was constructed and identified using Cufflinks [20]. Gene expression was quantified using fragments per kilobase of transcript per million reads mapped (FPKM). Finally, differentially expressed genes were obtained by paired t-test with false discovery rate (FDR) < 0.05.

### Prediction of lncRNAs from RNA-seq data of lung tumor tissues

To predict novel lncRNAs in lung cancer, we downloaded the RNA-seq binary sequence alignment map (BAM) files of 485 lung adenocarcinoma tissues and 56 adjacent normal tissues from The Cancer Genome Atlas (TCGA). Our process of predicting lncRNAs followed a previously described workflow with modifications [21]. Briefly, transcripts with single exon or length < 160 bp were filtered. The protein coding potential of the remaining transcripts was evaluated by PhyloCSF based on the alignment with genomes of chimp, rhesus, mouse, guinea, pig, cow, horse and dog [22]. The transcripts with PhyloCSF scores greater than 50 were removed for their high coding potential. Meanwhile, transcripts with complete branch length (CBL) > 0 and open reading frame (ORF) of > 150 amino acids were removed. The transcripts with CBL scores equal to 0 due to poor sequence alignments were also removed if they contained an ORF with more than 50 amino acids. Finally, we used blastx with repeats masked to analyze the remaining transcripts and removed those with a median of the E-value <1e-18. The coding potential of the identified lncRNAs was further evaluated by CAPT [23] and CPC2 [24] with default parameters.

### Human lung cancer samples

To evaluate the expression of LCAT1, 25 paired lung adenocarcinoma tissues and corresponding adjacent normal lung tissues were obtained from the surgical specimen archives of the Sir Run Run Shaw Hospital of Zhejiang University (Additional file 3: Table S3). Tissue specimens were snap-frozen in liquid nitrogen and stored at − 80 °C for RNA extraction. Hematoxylin and eosin (H&E) slides was reviewed by a pathologist to confirm the diagnosis. The study was conducted in accordance with the International Ethical Guidelines for Biomedical Research Involving Human Subjects. All subjects provided informed consent to participate in the study.

### Analysis of copy number variation at the RAC1 locus

The level 3 data of the copy number variation (CNV) of the TCGA lung adenocarcinoma samples generated from Affymetrix SNP 6.0 was downloaded through GDC data portal (https://portal.gdc.cancer.gov/). The CNV value of RAC1 was defined as the segment mean whose absolute value was the largest among all segments covering RAC1 gene. The samples with CNV value less than 1 were retained for further analysis.

### Statistical analysis

For comparison of two groups, a two-tailed Student’s t test was used. Comparison of multiple groups were made using a one- or two-way ANOVA. All experiments were repeated at least 3 times, and representative experiments are shown. Difference was considered statistically significant at *P* < 0.05.

## Results

### LCAT1 is upregulated in lung cancer and is associated with poor prognosis

**Fig. 1:**
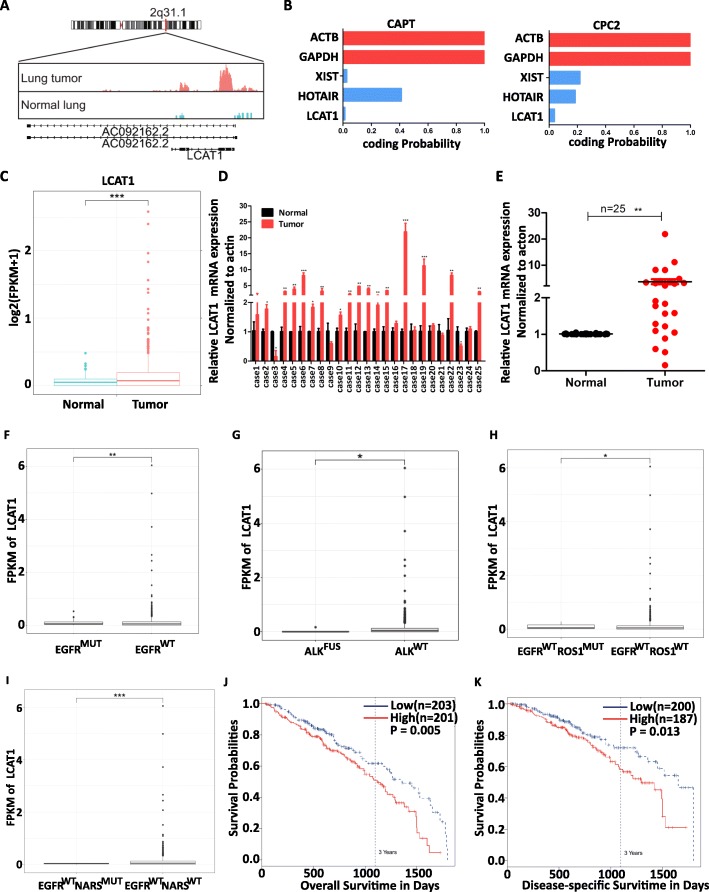
LCAT1 is upregulated in lung cancer tissues and is associated with poor prognosis. **a** Schematic representation of LCAT1 exons and transcripts and its loci on human chromosome 2q31.1. **b** The protein coding potential of LCAT1 evaluated by the Coding Potential Assessment Tool (CPAT) and Coding Potential Calculator 2 (CPC2). A smaller CAPT/CPC2 score indicates low probability of protein coding potential. **c** Relative expression of LCAT1 in lung tumor tissues and adjacent normal tissues in TCGA cohort. The expression of LCAT1 was quantified by fragments per kilobase of transcript per million reads mapped (FPKM) in the RNA sequencing data. **d, e** LCAT1 expression validated by qRT-PCR in an independent cohort of 25 pairs of lung cancer tissues and matched adjacent normal tissues. LCAT1 expression was normalized to the expression of actin. **f, g, h, i** Difference in the expression of LCAT1 between lung adenocarcinoma patients with somatic mutations in EGFR (**f**), ALK (**g**), ROS (**h**) or NRAS (**i**) and those without such mutations. **j, k** Kaplan-Meier curves of overall survival (**j**) and disease-specific survival (**k**) based on LCAT1 expression levels. Error bars indicate standard error of mean (SEM). Data are presented as mean ± SEM. *P < 0.05; **P < 0.01; ***P < 0.001

To identify the role of lncRNAs in lung carcinogenesis, we first analyzed the RNA-seq data of 485 lung adenocarcinoma tissues and 56 adjacent normal tissues from TCGA. In order to predict lncRNA, we used a previously described protocol, with minor modification [25]. We identified a novel lung cancer associated transcript 1 (LCAT1), which is located on human chromosome 2q31.1. LCAT1 has one transcript with three exons (Fig. 1a). Both Coding Potential Assessment Tool (CAPT) [23] and Coding Potential Calculator 2 (CPC2) [24] indicated that LCAT1 is a non-coding RNA and does not have protein-coding capacity (Fig. 1b). LCAT1 has not been annotated in the GENCODE (v29) database. The rapid amplification analysis of 5′ and 3′ cDNA ends (RACE) revealed that LCAT1 is 896 bp in length (Additional file 4: Figure S1A and B).

The expression level of LCAT1 was significantly higher in lung tumor tissues than in normal lung tissues (*P* < 0.001) (Fig. 1c). To validate this observation, we quantified the expression level of LCAT1 in independent lung cancer samples using quantitative PCR (qPCR), which confirmed increased LCAT1 expression in lung cancer tissues compared to adjacent normal tissues (Fig. 1d and e). Interestingly, LCAT1 was upregulated in a distinct subgroup of lung cancer patients who did not have actionable mutations in EGFR, ALK, ROS or NRAS. This implies that the upregulation of LCAT1 may harbor an oncogenic driver event in lung carcinogenesis (Fig. 1f–i).

Furthermore, to assess the clinical significance of LCAT1 overexpression in lung cancer, we evaluated the correlation between LCAT1 expression levels and clinical outcomes of patients. Kapan-Meier survival analysis revealed that the patients with higher levels of LCAT1 had shorter overall survival and disease-specific survival times than those with lower levels of LCAT1 (Fig. 1j and k).

### LCAT1 silencing inhibits lung cancer cell proliferation in vitro and in vivo

**Fig. 2:**
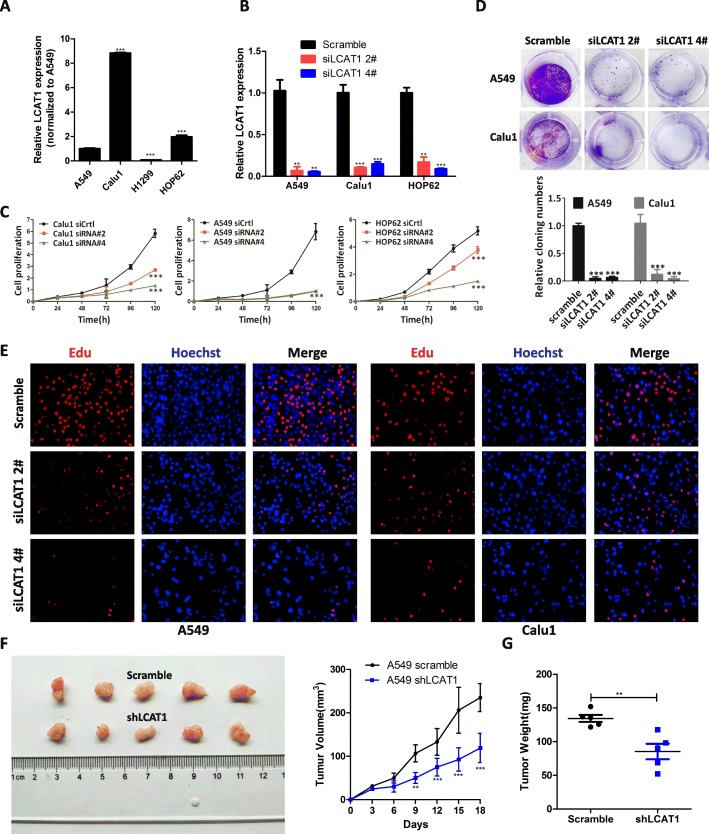
LCAT1 promotes lung cancer cell proliferation in vitro and in vivo. **a** qRT-PCR analysis of LCAT1 expression in lung cancer cells. **b** Relative expression of LCAT1 in lung cancer cells transfected with LCAT1 siRNA (si-LCAT1 2# or si-LCAT1 4#) and scrambled siRNA. **c** Growth curve of lung cancer cells transfected with LCAT1 siRNA or scrambled siRNA by CCK-8 assays. **d, e** Proliferation of lung cancer cells transfected with LCAT1 siRNA or scrambled siRNA as determined by colony formation assay (**d**) and EdU staining assay (**e**). **f, g** Tumor volume and weight of mouse xenografts subcutaneously injected with A549 cells with stable LCAT1 knockdown. The tumor growth curve was measured every 3 days. Nude mice were euthanized 3 weeks following treatment and the tumor nodules were collected. All in vitro experiments were performed in triplicate and one of representative results was presented. Error bars indicate standard error of mean (SEM). Data are presented as mean ± SEM. *P < 0.05; **P < 0.01; ***P < 0.001. The below is same for other figures

To explore the biological function of LCAT1 in the lung cancer cells, we measured its expression level in four lung cancer cell lines (A549, Calu1, H1299, and HOP62) using qPCR. LCAT1 was widely expressed in these lung cancer cell lines (Fig. 2a). We knocked down or overexpressed LCAT1 in lung cancer cells by transfecting them with siRNA, or with an overexpression plasmid (pENTER-LCAT1), respectively (Fig. 2b and Additional file 5: Figure S2A). The growth curves obtained from CCK8 proliferation assay indicated that LCAT1 knockdown significantly inhibited cell proliferation and colony formation in the A549, Calu1, and HOP62 cell lines (Fig. 2c and d, and Additional file 5: Figure S2C). Further, LCAT1 overexpression promoted cell growth ability in the H1299 and A549 (Additional file 5: Figure S2B) cell lines. Additionally, the 5-ethynyl-2′-deoxyuridine (EdU) proliferation assay confirmed that the depletion of LCAT1 resulted in reduced cell growth (Fig. 2e and Additional file 5: Figure S2D).

To further examine the function of LCAT1 in vivo, we subcutaneously injected A549 and Calu1 cells with stable knockdown of LCAT1 into the nude mice. The volume and weight of tumor in the shLCAT1 (LCAT1 knockdown) group were significantly smaller than the control group (Fig. 2f and g, and Additional file 5: Figure S2E and F), suggesting that LCAT1 can promote tumorigenicity of lung cancer cells in vivo.

### LCAT1 knockdown decreases metastasis and induces G1 arrest of lung cancer cells

**Fig. 3:**
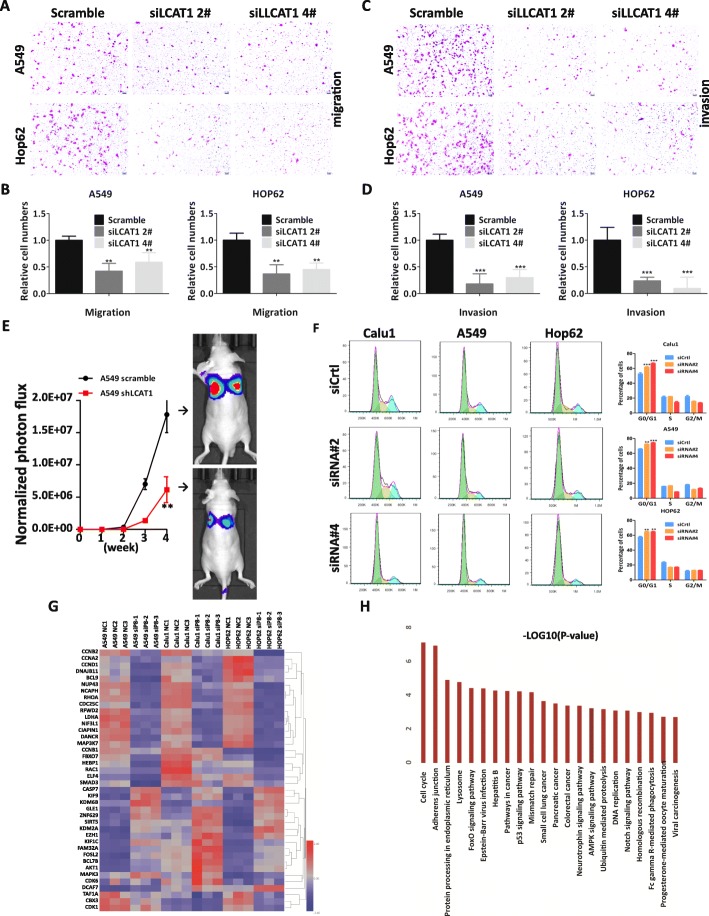
Effects of LCAT1 on lung cancer cell migration, invasion, and cell cycle. **a-d** Migration and invasion ability of A549 and HOP62 cells transfected with LCAT1 siRNA or scrambled siRNA. **e** LCAT1 promotes lung cancer metastasis in vivo. **f** Cell cycle profile of control and si-LCAT1 lung cancer cells by flow cytometry. **g** Hierarchical clustering of transcripts altered in lung cancer cells after LCAT1 knockdown. **h** GSEA enrichment analysis of signaling pathways and biological process enriched for differentially expressed transcripts after LCAT1 knockdown

LCAT1 knockdown significantly reduced the migration and invasion of lung cancer cells (Fig. 3a–d, and Additional file 6: Figure S3A–C). To further explore the metastasis-promoting effect of LCAT1 on lung cancer cells in vivo, we intravenously injected stable LCAT1 knockdown cells into the lateral tail veins of nude mice. We observed that knockdown of LCAT1 significantly reduced lung cancer cell metastasis in vivo (Fig. 3e). Furthermore, a flow cytometry assay was performed to analyze the effect of LCAT1 knockdown on the cell cycle of lung cancer cells. We found that transfection with two independent LCAT1-specific siRNAs (si-LCAT1 2# or siLCAT1 4#) induced G1 arrest in lung cancer cells (Fig. 3f). Consistent with the cell cycle progression phenotype, the expression level of cell-cycle related proteins, Cyclin D1, Cyclin A2 and Cyclin B, was decreased when LCAT1 expression was knocked down (Additional file 6: Figure S3D).

To investigate the related signaling pathways in the lung cancer cells, an unbiased transcriptome profiling of the three cell lines transfected with si-LCAT1 or scramble siRNA was performed using RNA-seq technology (Additional file 2: Table S2). Differential expression patterns were observed between the LCAT1 knockdown cells and scrambled controls (Fig. 3g). The gene set enrichment analysis (GSEA) revealed that these differentially expressed genes participate in many important signaling pathways. The major targeted pathways included cell cycle and adhesion junction (Fig. 3h).

### LCAT1 functions as a ceRNA and sponges miR-4715-5p in lung cancer cells

**Fig. 4:**
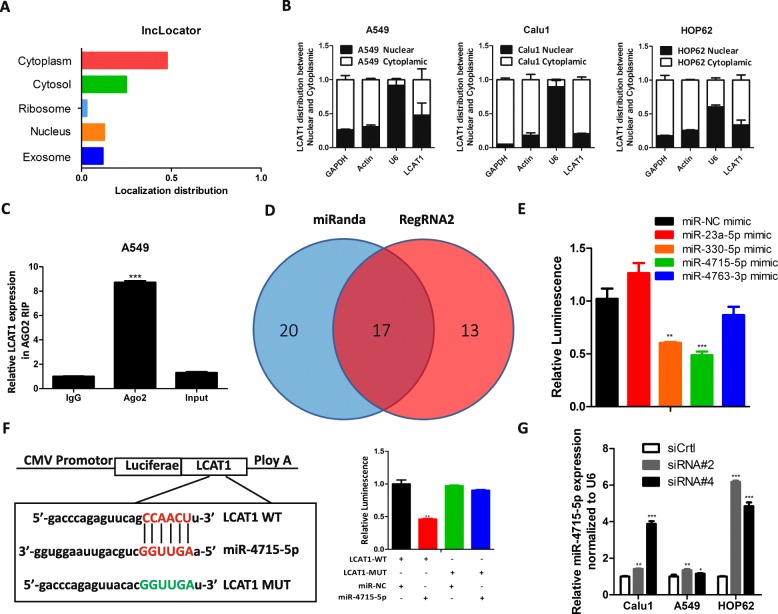
LCAT1 interacts with miR-4715-5p in lung cancer cells. **a, b** LCAT1 localization was predicted using lncRNA subcellular localization predictor, lncLocator (**a**) and subcellular fractionation (**b**). **c** RIP experiments were performed in A549 cells and the coprecipitated RNA was used to quantify LCAT1 expression using qRT-PCR. **d, e** Potential miRNA binding to LCAT1. miRNA-LCAT1 interaction was predicted by miRanda and RegRNA2, of which 17 were overlapped. Several common miRNAs were further validated using a luciferase reporter assay. **f** Luciferase reporter assay shows that miR-4715-5p binds to LCAT1. The luciferase reporter plasmid containing wildtype (Wt) or mutant (Mut) LCAT1 was co-transfected into A549 cells with miR-4715-5p mimics or miR-NC mimics. **g** miR-4715-5p expression by qPCR in lung cancer cells transfected with control siRNA or si-LCAT1. U6 was used as an internal control gene for the quantification of miRNA expression

Accumulating evidence has indicated that lncRNAs can regulate target gene expression by interacting with RNA-binding proteins, or by functioning as a ceRNA for microRNA (miRNA) [26–34]. To determine the molecular mechanism by which LCAT1 promotes the lung cancer cell proliferation and metastasis, we first predicted its localization in cells using lncRNA subcellular localization predictor (lncLocator, http://www.csbio.sjtu.edu.cn/bioinf/lncLocator/) software and subcellular fractionation [35]. Both of these data suggested that LCAT1 was mainly localized to the cytoplasm (Fig. 4a and b, and Additional file 7: Figure S4A), indicating that LCAT1 might regulate target protein expression at the posttranscriptional level. Furthermore, an immunoprecipitation assay for RNA binding protein in the A549 extracts revealed that LCAT1 binds to Ago2, a main component of the RNA-induced silencing complex that is involved in the miRNA-mediated repression of messenger RNA (mRNA) (Fig. 4c). These results indicated that LCAT1 may function as a ceRNA of miRNAs.

To test this hypothesis, we used two bioinformatics databases (miRanda and RegRNA2) to predict the potential interaction between miRNAs and LCAT1. We found that LCAT1 contains multiple miRNA binding sites (Fig. 4d). We then prioritized these miRNAs according to their prediction score and free-energy, and chose the top four miRNAs (miR-23a-5p, miR-330-5p, miR-4715-5p and miR-4763-3p) for subsequent dual luciferase reporter assays. The A549 cells were transfected with a luciferase plasmid containing the sequence of LCAT1 along with miRNA mimics or control mimics. Among them, only miR-330-5p and miR-4715-5p mimics could suppress LCAT1-driven luciferase activity, and the inhibition of miR-4715-5p was stronger than miR-330-5p (Fig. 4e). Therefore, we pursued miR-4715-5p as a primary candidate for further investigation. We designed a reporter construct in which the putative miR-4715-5p-binding site in the LCAT1 sequence was mutated by site-directed mutagenesis. As expected, the suppression of miR-4715-5p was abolished (Fig. 4f).

Next, we evaluated the expression level of miR-4715-5p in lung cancer cell lines after silencing or overexpressing LCAT1. There was a weak inverse correlation between miR-4715-5p and LCAT1 in these cancer cells (Additional file 7: Figure S4B). LCAT1 knockdown significantly increased miR-4715-5p expression (Fig. 4g). In contrast, overexpression of LCAT1 slightly reduce miR-4715-5p expression (Additional file 7: Figure S4C). Reciprocally, LCAT1 expression was also decreased in cells overexpressing miR-4715-5p (Additional file 7: Figure S4D). However, miR-4715-5p was not significantly associated with patients’ survival in TCGA lung cancer samples (Additional file 7: Figure S4E and F).

### LCAT1 function is partially mediated by repressing miR-4715-5p

**Fig. 5:**
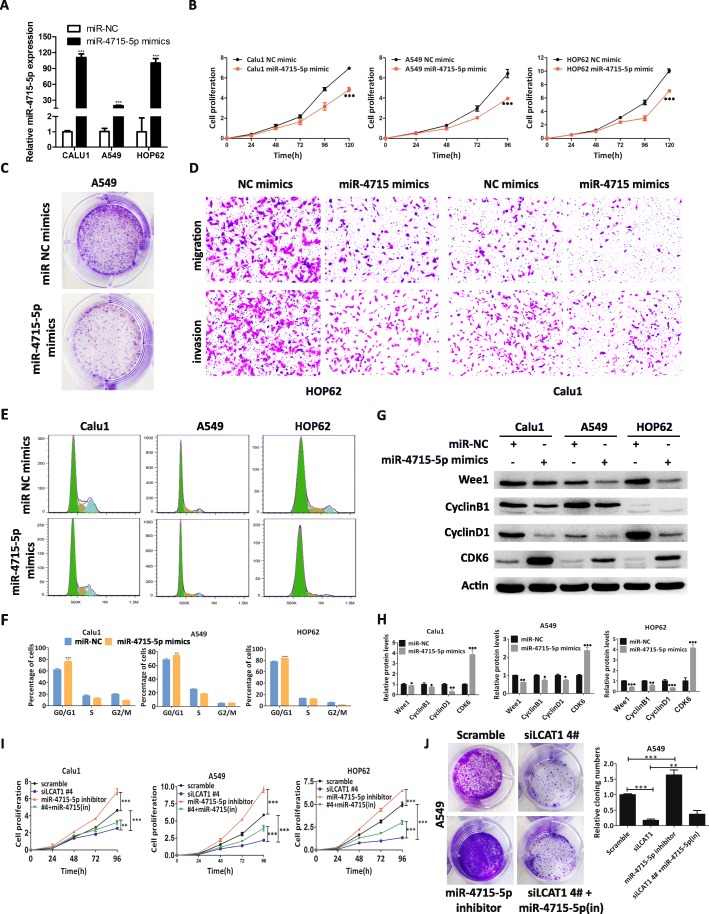
miR-4715-5p inhibits lung cancer cell proliferation, migration, invasion, and cell cycle. **a** miR-4715-5p expression in lung cancer cells transfected with control miRNA or miR-4715-5p mimics was quantified by qRT-PCR. **b, c** The inhibitory effect of miR-4715-5p mimic on cell proliferation was evaluated using a CCK8 assay and a colony formation assay. **d** Migration and invasion ability of lung cancer cells transfected with control miRNA or miR-4715-5p mimics. **e, f** Cell cycle profile of lung cancer cells transfected with control miRNA or miR-4715-5p mimics. **g, h** Western blot analysis of cell cycle-related proteins in cells overexpressing miR-4715-5p. **i**, **j** Growth curve and colony formation in lung cancer cells co-transfected with si-LCAT1 and miR-4715-5p inhibitor

To investigate the function of miR-4715-5p in lung cancer cells, we transfected miR-4715-5p mimic to lung cancer cells and conducted CCK8, colony formation, and EdU assays accordingly (Fig. 5a). We found that miR-4715-5p overexpression could significantly decrease cell proliferation and colony formation in lung cancer cells (Fig. 5b and c, and Additional file 8: Figure S5A and B). In contrast, lung cancer cell proliferation was enhanced by silencing miR-4715-5p expression (Additional file 8: Figure S5C). Moreover, the migration and invasion were also reduced in lung cancer cells overexpressing miR-4715-5p (Fig. 5d and Additional file 8: Figure S5D). Flow cytometry analysis indicated that overexpression of miR-4715-5p induced cell-cycle arrest at G1-G0 phase in the Calu1, A549 and HOP62 cell lines (Fig. 5e and f). Subsequently, Western blot analysis revealed that the expression levels of the cell cycle-related proteins, cyclin D1 and cyclin B1, were downregulated, whereas CDK 6 protein expression was upregulated (Fig. 5g and h).

To study whether miR-4715-5p mediates the function of LCAT1 in lung cancer cells, we co-transfected si-LCAT1 with a miR-4715-5p inhibitor into lung cancer cells. siLCAT1-mediated inhibition of the cell growth was partially rescued by co-transfection with the miR-4715-5p inhibitor (Fig. 5i and j, and Additional file 8: Figure S5E), suggesting that LCAT1 promotes cell proliferation, at least in part, by repressing miR-4715-5p function.

### RAC1 is a target gene of miR-4715-5p and is indirectly regulated by LCAT1

**Fig. 6:**
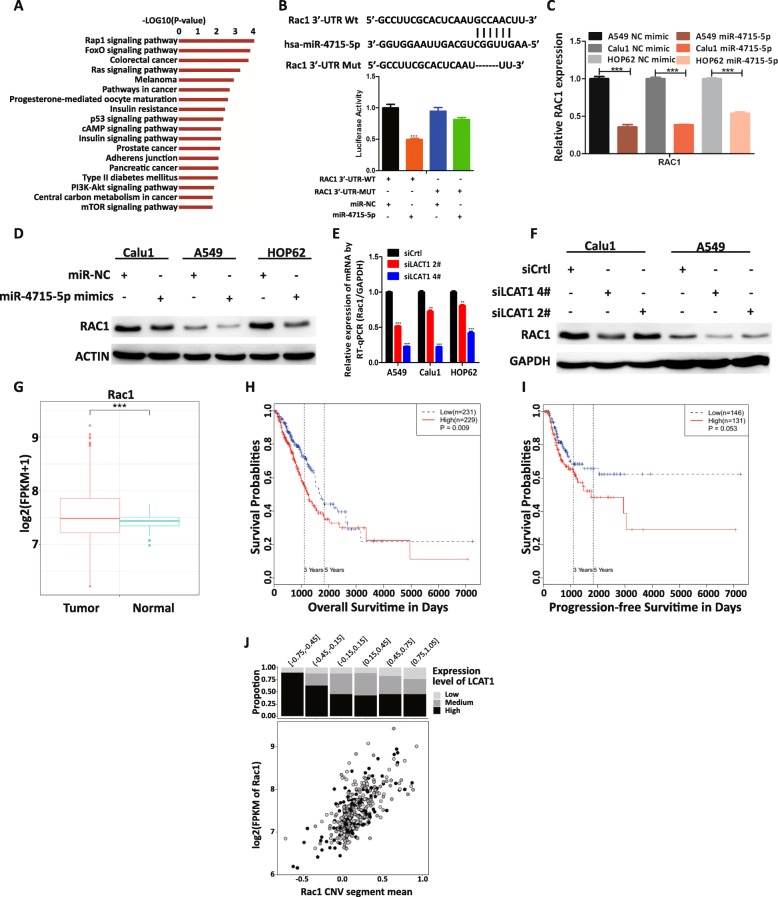
RAC1 is a target of miR-4715-5p and indirectly regulated by LCAT1. **a** GSEA enrichment analysis of miR-4715-5p potential targets. **b** Luciferase activity assay in A549 cells transfected with plasmid containing RAC1 3’UTR (wildtype, Wt or mutant, Mut) and miR-4715-5p mimics or control miRNA. **c** RAC1 expression in lung cancer cells transfected with control miRNA or miR-4715-5p mimics. **d** Western blot analysis of RAC1 expression in the lung cancer cells transfected with control miRNA or miR-4715-5p mimics. **e, f** RAC1 mRNA and protein levels in lung cancer cells following LCAT1 knockdown. **g** RAC1 expression in lung tumor tissues and adjacent normal tissues from TCGA cohort. **h, i** Kaplan-Meier survival analysis of overall survival and progression-free survival time in lung cancer patients based on RAC1 expression. **j** Relationship between LCAT1 expression, RAC1 expression and RAC1 copy number variation (CNV)

To investigate the ceRNA network among LCAT1, miR-4715-5p, and its targets in lung cancer, we used TargetScanHuman (http://www.targetscan.org/vert72/) with default parameters to predict potential miR-4715-5p target genes. Next, we combined these predictions with our RNA-seq data from LCAT1 knockdown and control cell lines to prioritize the predicted targets. Specifically, we performed GSEA analysis on miR-4715-5p target genes and revealed that these target genes are involved in many signaling pathways, including the Ras signaling pathway (Fig. 6a). Additionally, RAC1 was one of the most downregulated genes in LCAT1 knockdown cells. Thus, RAC1 was chosen as the primary target for further investigation. To further validate our selected target, we performed luciferase reporter assays driven by the wild-type 3′-untranslated (UTR) sequence of RAC1, which contains potential miR-4715-5p binding sites (RAC1-Wt), or mutant constructs containing a mutation in the miR-4715-5p binding sites (RAC1-Mut). These plasmids were co-transfected into the A549 cells together with miR-4715-5p mimic or negative control mimic. We observed that only the miR-4715-5p mimic could repress the RAC1-Wt-driven luciferase activity, but not negative control mimic or RAC1-Mut-driven luciferase activity (Fig. 6b).

To further determine whether RAC1 is regulated by miR-4715-5p in lung cancer cells, we measured RAC1 mRNA and protein levels in lung cancer cells with depletion or overexpression of miR-4715-5p. We observed that RAC1 mRNA and protein levels were significantly downregulated in cells overexpressing miR-4715-5p (Fig. 6c and d). We also quantified the expression levels of LCAT1/miR-4715-5p/RAC1 using qPCR in our lung cancer tissues. There was a weak positive correlation between LCAT1 and RAC1 (Additional file 9: Figure S6A), and a negative correlation between miR-4715-5p and LCAT1/RAC1 in the lung cancer tissues (Additional file 9: Figure S6B and C). Taken together, these data suggested that miR-4751-5p regulates RAC1 expression in lung cancer cells by directly binding to 3′ UTR of RAC1 mRNA.

Since LCAT1 can sponge miR-4715-5p, we next determined whether LCAT1 could affect the expression of RAC1 through competitive binding with miR-4715-5p. We found that LCAT1 knockdown significantly reduced the RAC1 mRNA and protein levels in the Calu1 and A549 cells (Fig. 6e and f). To investigate the role of miR-4715-5p with LCAT1 and RAC1, we measured the RAC1 expression in cells co-transfected with si-LCAT1 and miR-4715-5p inhibitor. As expected, the RAC1 expression was partially restored in co-transfected cells (Additional file 9: Figure S6D).

We analyzed RAC1 expression in the RNA-seq data of lung cancer and normal tissues from the TCGA cohort. The results demonstrated that RAC1 is significantly upregulated in lung cancer tissues (Fig. 6g). Moreover, higher levels of RAC1 was significantly associated with poor prognosis of lung cancer patients (Fig. 6h and i).

Furthermore, there was no somatic mutation detected in RAC1 in lung adenocarcinomas from TCGA cohort, whereas increased copy number was observed at the RAC1 locus in lung adenocarcinomas. The copy number of RAC1 was strongly and positively correlated with its mRNA expression. However, it is worth noting that lung cancer patients with normal or low copy number of RAC1 tended to have a higher expression of LCAT1 that could potentially drive the expression of RAC1 (Fig. 6j).

### LCAT1 promotes lung cancer cell proliferation and progression through sponging miR-4715-5p to regulate RAC1/PAK1 functions

**Fig. 7:**
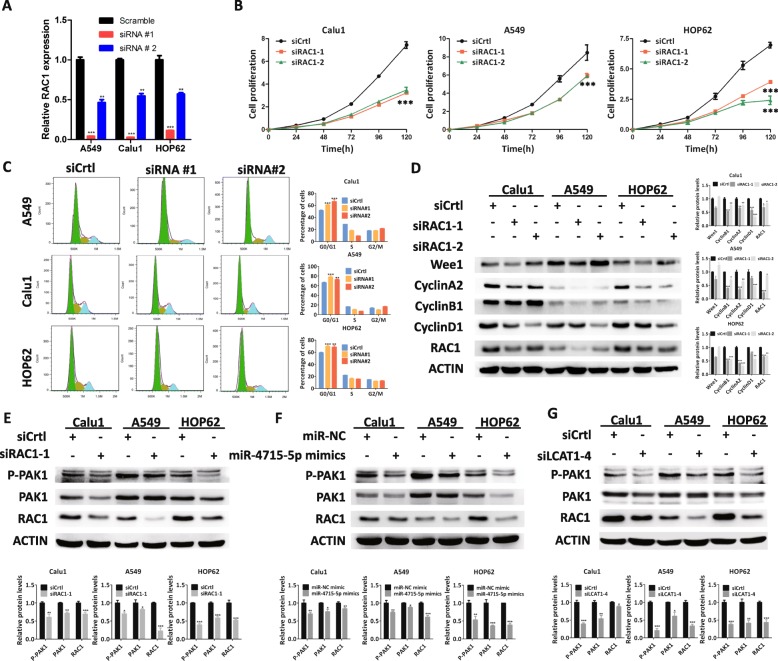
RAC1 promotes lung cancer cell proliferation and cell cycle. **a** RAC1 expression in lung cancer cells transfected with RAC1 siRNA or scramble siRNA. **b** Cell proliferation following RAC1 knockdown was evaluated using CCK8 assay. **c** Cell cycle profile of lung cancer cells transfected with RAC1 siRNA or scramble siRNA. **d** Western blot analysis of cell-cycle related proteins in cells transfected with RAC1 siRNA or scrambled siRNA. **e**-**g** PAK1 expression in RAC1 suppressed, miR-4715-5p overexpressed, or LCAT1 silenced lung cancer cells

To determine the biological function of RAC1 in lung cancer cells, we used siRNA to knockdown RAC1 in the Calu1, A549 and HOP62 cell lines (Fig. 7a). CCK8 and colony formation assays indicated that the knockdown of RAC1 expression significantly inhibited lung cancer cell proliferation and colony formation (Fig. 7b, and Additional file 10: Figure S7A and S7B). Transwell migration and invasion assay revealed that the knockdown of RAC1 remarkably reduced cell migration and invasion (Additional file 10: Figure S7C). The proliferation defects associated with LCAT1 knockdown could also be rescued by overexpression of RAC1 (Additional file 10: Figure S7D). Furthermore, the knockdown of RAC1 induced cell-cycle arrest at G1-G0 phase which coincided with both the inhibition of LCAT1 and overexpression of miR-4715-5p (Fig. 7c). This was also confirmed by Western blot analysis, which showed that RAC1 knockdown decreased protein levels of Cyclin D1, Cyclin B1 and Cyclin A1 (Fig. 7d).

Previous studies have demonstrated that RAC1 is a member of small GTPases and plays an important role in modulating cellular functions. Furthermore, PAK1 (p21-activated kinase 1) is a down-stream effector of RAC1 and partially mediates RAC1 function [36]. Hence, we hypothesized that RAC1 could regulate PAK1 phosphorylation to affect the cell function. Using Western blot, we observed that phosphorylated PAK1 expression was significantly reduced when RAC1 was inhibited (Fig. 7e). Consistently, phosphorylated PAK1 expression was downregulated in both the miR-4715-5p overexpressed and the LCAT1 knockdown lung cancer cells (Fig. 7f and g).

### RAC1 inhibitor and paclitaxel combination for lung cancer

**Fig. 8:**
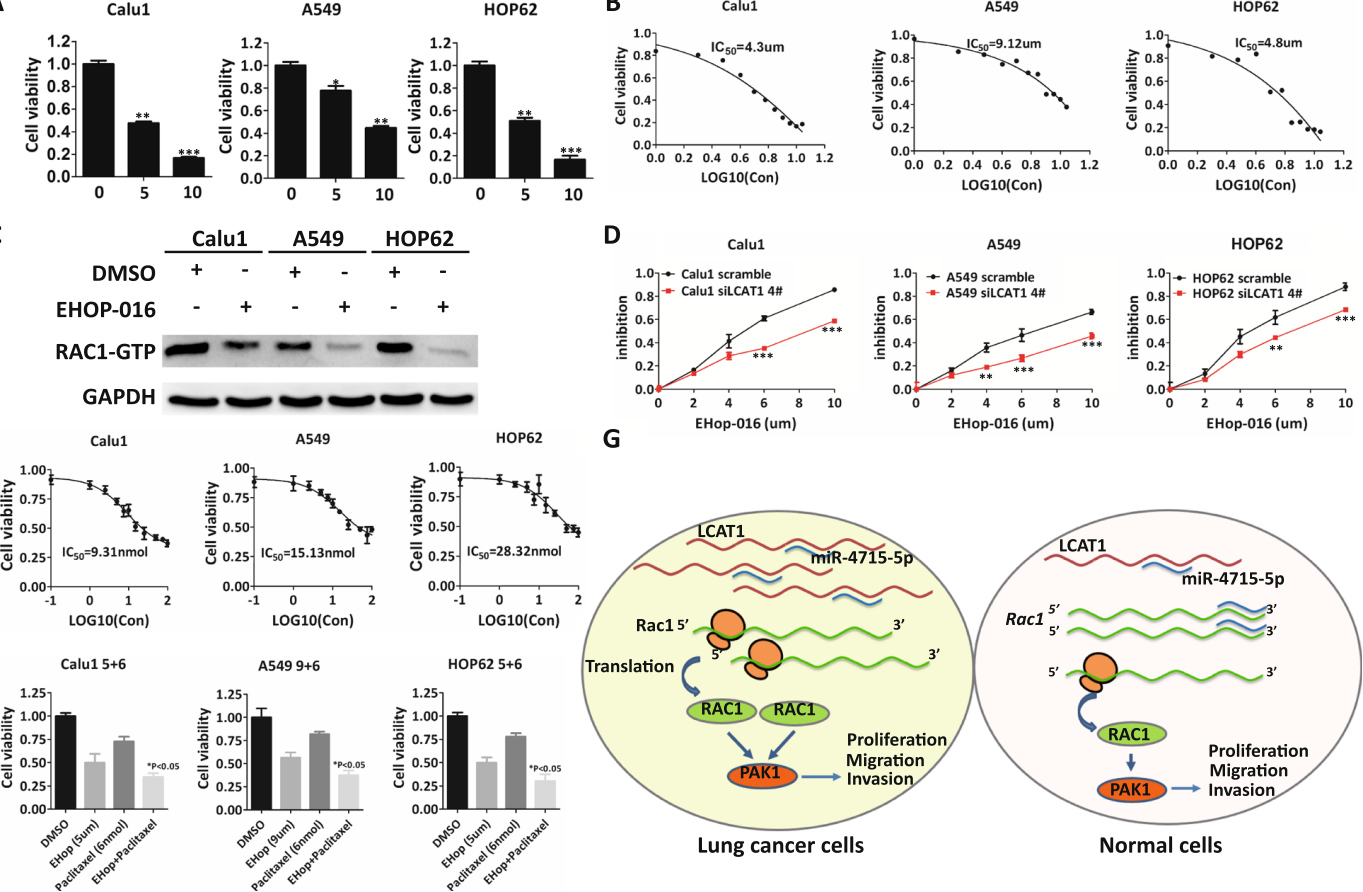
Efficacy of RAC1 inhibitor and paclitaxel combination therapy in lung cancer cells. **a** Cell viability in the presence of increased concentration of EHop-016, a RAC1 inhibitor. **b** Growth curve of lung cancer cells treated with different concentrations of EHop-016 (1.0–5 μmol/L for 48 h). IC_50_ of EHop-016 for different lung cancer cell lines was determined according to their growth curve. **c** Western blot analysis of active RAC1 in lung cancer cells after treatment with EHop-016. **d** The inhibition efficacy of siLCAT1 and control cells under different concentrations of EHop-016. Cell viability was used to evaluate the inhibition efficacy of EHop-016. **e** Cell viability after paclitaxel treatment (0.1–100 nmol/L for 48 h). **f** Efficacy of EHop-016 (5.0 μmol/L for Calu1 and HOP62, 9 μmol/L for A549) and paclitaxel (6 nmol/L) combination therapy in lung cancer cells. The cell viability of lung cancer cells was measured after treatment for 48 h using CCK8 assays. **g** Proposed model for LCAT1-mediated regulation of proliferation, migration, and invasion in lung cancer cells

Our data has demonstrated that genetic targeting of RAC1 by siRNA causes a significant reduction in cell proliferation and metastasis of lung cancer cells (Fig. 7 and Additional file 10: Figure S7). We thus speculated whether a similar outcome could be achieved through pharmacological inhibition of RAC1 activity. EHop-016 is a novel small molecule inhibitor of RAC GTPase [37]. EHop-016 treatment can not only reduce RAC1 activity but also inhibit the RAC1 downstream effects of PAK1 activity [37]. By employing CCK8 assay, we found that EHop-016 could inhibit lung cancer cell proliferation (Fig. 8a). The IC_50_ of EHop-016 for Calu1, A549, and HOP62 cell lines was 4.3, 9.12, and 4.8 μmol/L, respectively (Fig. 8b). Next, we measured the impact of EHop-016 treatment (low dose of 2.0 μmol/L in the Calu1 and HOP62 cell lines, 4.5 μmol/L in the A549 cell line for 48 h) on active RAC1. We observed that EHop-016 could reduce RAC1-GTP expression (Fig. 8c), while LCAT1 knockdown de-sensitized the cells to EHop-016 treatment (Fig. 8d).

Paclitaxel is a chemotherapy drug widely used for treating many malignancies, including lung cancer. Therefore, we investigated the efficacy of RAC1 inhibitor-based combination therapy in vitro. By employing CCK8 assay, we determined the efficacy of paclitaxel on the growth of lung cancer cells and measured IC_50_. The IC_50_ of paclitaxel against the Calu1, A549 and HOP62 cell lines was estimated to be 9.31, 15.13 and 28.32 nmol/L, respectively (Fig. 8e). Notably, the combination treatment of EHop-016 and paclitaxel resulted in 60–70% reduction in cell growth, which exhibited a significantly larger impact on the cell viability (*P* < 0.05) than EHop-016 and paclitaxel monotherapies (Fig. 8f).

## Discussion

Emerging studies have shown that lncRNAs play a critical role in cancer [38–40]. Although a large number of lncRNAs have been identified in the human genome, only a very few have been experimentally validated and functionally annotated in lung cancer [41–44]. In the present study, we identified a novel lncRNA, LCAT1, which is markedly upregulated in lung cancer tissues. Importantly, higher expression of LCAT1 is highly predictive of the shorter survival in patients with lung cancer, suggesting that LCAT1 is a potential prognostic biomarker for lung cancer. Both in vitro and in vivo assays demonstrated that LCAT1 exhibited strong oncogenic activity by promoting lung cancer cell proliferation, migration, and invasion.

Increasing evidence suggests the existence of a widespread interaction network involving ceRNAs, in which ncRNAs could regulate target RNA by binding and titrating them off their binding sites on protein coding messengers [45]. LncRNA functions are closely related to their subcellular localization. In the present study, we determined that LCAT1 is mainly localized to the cytoplasm and interacts with Ago2 in lung cancer cells, suggesting that LCAT1 may function as an endogenous miRNA sponge. Bioinformatics analysis and luciferase reporter assays revealed that miR-4715-5p is a target of LCAT1. In addition, the expression of miR-4715-5p was enhanced in lung cancer cells upon LCAT1 knockdown, which confirmed our hypothesis. However, the function of miR-4715-5p in cancer is rarely studied. In this study, we demonstrated that overexpressing miR-4715-5p in lung cancer cell lines could inhibit cell proliferation and induce cell-cycle arrest at G1-G0 phase. Our findings revealed the significance of the interaction between LCAT1 and miR-4715-5p in lung tumorigenesis given that LCAT1 exerts oncogenic function partly via sponging miR-4715-5p in lung cancer cells.

We first used bioinformatics analysis to predict miR-4715-5p targets, followed by validation using a luciferase assay. We found that RAC1 was the strongest target and was directly regulated by miR-4715-5p. Whereas the expression level of miR-4715-5p was affected by the endogenous level of LCAT1. RAC1 is widely expressed in human tissues and regulates cell proliferation and cell motility [46, 47]. Overexpression of RAC1 is involved in multiple human cancers such as breast cancer and liver cancer [48, 49]. Therefore, we assumed that LCAT1 could modulate RAC1 mRNA level by competitively sponging miRNA-4715-5p, thereby enhancing lung cancer cell proliferation and invasion. Consistent with our hypothesis, we found that RAC1 expression was upregulated in lung cancer tissues compared to normal tissues. Higher RAC1 expression were significantly associated with poor prognosis in lung cancer patients. Depletion of RAC1 inhibited cell proliferation and motility. Moreover, we identified its downstream target, PAK1, which partly mediated RAC1 function. Both RAC1 and PAK1 were downregulated in cells overexpressing miR-4715-5p and in LCAT1 knockdown cells.

Interestingly, increased copy number was observed at the RAC1 locus in lung cancer, which consequently upregulated its mRNA expression. We also observed that lung cancer patients with normal or low copy number of RAC1 tended to have higher LCAT1 expression. These data suggested that two mechanisms potentially mediate the RAC1 expression in lung cancer: one is through genomic amplification which causes elevated RAC1 expression and the other is through the upregulation of LCAT1 which competitively sponges miRNA-4715-5p to regulate the RAC1 mRNA expression. In the second mechanism, miR-4715-5p serves as a mediator between LCAT1 and RAC1. Overexpression of LCAT1 in lung tumor can sponge more miR-4715-5p and thus leads to less miRNA-mediated mRNA decay of RAC1 by miR-4715-5p. This promotes the aggressive growth of tumor and ultimately influences patients’ survival (Fig. 8g). We estimated pairwise correlations among three genes in our lung cancer tissues. There was a weak positive correlation between LCAT1 and RAC1, and a negative correlation between miR-4715-5p and LCAT1/RAC1 in lung cancer tissues (Additional file 9: Figure S6). These trends are in good agreement with our proposed model (Fig. 8g).

Finally, our data demonstrated that EHop-016, a small molecule inhibitor of Rac GTPase, could decrease lung cancer cell viability. The combination of EHop-016 and paclitaxel exhibited better efficacy than the respective monotherapy for treating lung cancer cells. This suggests that EHop-16 could potentially be used as an adjuvant to improve the therapeutic effect of paclitaxel in lung cancer patients with high expression of LCAT1. It is also worth noting that LCAT1 was upregulated in a distinct subgroup of lung cancer patients that don’t have actionable mutations in EGFR, ALK, ROS or NRAS. In other words, there is a lack of targeted therapy in this subgroup of lung cancer patients characterized by LCAT1 overexpression. Therefore, there is an urgent need to develop an effective combination therapy of RAC1 inhibitor and paclitaxel for this subgroup of cancer patients.

In conclusion, our study identified a novel lncRNA associated with poor prognosis in lung cancer. LCAT1 is an oncogenic regulator that promotes cell proliferation and metastasis. It induces competitive binding with miR-4715-5p, resulting in the upregulation of RAC1 and PAK1 (Fig. 8g). LCAT1 overexpression defines a distinct subgroup in lung cancer patients with poor prognosis. This subgroup of patients usually don’t have actionable mutations in EGFR, ALK, ROS or NRAS. Thus, there is a lack of targeted therapy in the subgroup of lung cancer patients characterized by LCAT1 overexpression. Our findings suggest that the LCAT1-miR-4715-5p-RAC1/PAK1 axis could be a valuable target for lung cancer prognosis and therapies. The novel strategy for treating lung cancer using a combination of paclitaxel and EHop-016 warrants further investigation, especially in the subgroup of lung cancer patients exhibiting LCAT1 overexpression.

## Supplementary information

**Additional file 1: Table S1.** List of antibodies and primers used in this study.

**Additional file 2: Table S2.** Quality control metrics of the RNA sequencing libraries.

**Additional file 3: Table S3.** Characteristics of lung cancer patient samples used in the study.

**Additional file 4: Figure S1.** RACE analysis and sequence of LCAT1. (A) Images of PCR products from the 5′ RACE and 3′ RACE. (B) Full-length sequence of LCAT1.

**Additional file 5: Figure S2.** LCAT1 affects lung cancer cell proliferation. (A) Overexpression of LCAT1 in A549 and H1299 cell lines. (B) CCK8 assay was used to determine the proliferation of cells overexpressing LCAT1. (C, D) Colony formation assay and EdU assay were performed in Calu1 cells. (E, F) Tumor volume and weight of mouse xenografts subcutaneously injected with Calu1 cells with stable LCAT1 knockdown. The tumor growth curve was measured every 3 days. Nude mice were euthanized 3 weeks following treatment and the tumor nodules were collected. All in vitro experiments were performed in triplicate and one of representative results was presented. Values are expressed as mean ± SEM, *P < 0.05; ** *P* < 0.01; ***P < 0.001. The below is same for other figures.

**Additional file 6: Figure S3.** LCAT1 promotes lung cancer cell migration and invasion. (A) Representative images of transwell migration and invasion assay and **(**B) Number of cells between si-LCAT1 and scrambled control. (C) Transwell migration and invasion assay for A549 cells transfected with si-LCAT1. (D) Western blot analysis of cell cycle-related proteins after transfection with control siRNA, si-LCAT1 2#, or si-LCAT1 4# in the Calu1, A549 and HOP62 cells. Actin protein was used as an internal control.

**Additional file 7: Figure S4.** The negative correlation between LCAT1 and miR-4715-5p. (A) Western blot analysis of subcellular fraction proteins. (B) Quantification of LCAT1, miR-4715-5p, and RAC1 expression by qPCR in lung cancer cell lines. (C) Quantification of miR-4715-5p expression in the cells overexpressing LCAT1 by qRT-PCR. **(**D) Quantification of LCAT1 expression by qRT-PCR in cells overexpressing miR-4715-5p. (E, F) Kaplan-Meier survival analysis of overall survival and progression-free survival time in lung cancer patients based on miR-4715-5p expression.

**Additional file 8: Figure S5.** miR-4715-5p affects lung cancer cell proliferation and progression. (A) EdU assay was performed to quantify the proliferation of cells overexpressing miR-4715-5p. (B) Colony formation of cells overexpressing miR-4715-5p. (C) miR-4715-5p silencing promotes lung cancer cell proliferation. (D) Representative images of cells overexpressing miR-4715-5p from transwell migration and invasion assay. (E) Proliferation of Calu1 cells after co-transfected with control si-LCAT1–4# and miR-4715-5p inhibitor was measured using a colony formation assay.

**Additional file 9: Figure S6.** Relationship of LCAT1/miR-4715-5p/RAC1 in lung cancer tissues. (A, B, C) Pairwise correlations among LCAT1/miR-4715-5p/RAC1 in lung cancer tissues and adjacent normal tissues. The expression level of LCAT1/miR-4715-5p/RAC1 was measured by qPCR. (D) RAC1 expression in Calu1 cells transfected with si-LCAT1and miR-4715-5p inhibitor, respectively, and co-transfected with si-LCAT1and miR-4715-5p inhibitor. RAC1 expression was measured by Western blot.

**Additional file 10: Figure S7.** RAC1 affects lung cancer cell proliferation, invasion, and migration. (A, B) Proliferation of RAC1 knockdown cells measured by EdU and colony formation assays. (C) Migration and invasion ability of RAC1 knockdown Calu1 and HOP62 cells by transwell migration and invasion assay. (D) Proliferation of Calu1 and HOP62 cells co-transfected with siLCAT1 and pENTER-RAC1 plasmid measured by CCK-8 assay.

## Data Availability

All data that support the findings of this study are available from the corresponding authors upon reasonable request.

## Abbreviations

ATCC: American Type Culture Collection
CAPT: Coding Potential Assessment Tool
CCK8: a cell counting kit-8
cDNA: Complementary DNA
ceRNA1: Competing endogenous RNA
CPC2: Coding Potential Calculator 2
GSEA: Gene Set Enrichment Analysis
LCAT1: Lung Cancer Associated Transcript 1
LncLocator: LncRNA subcellular localization predictor
lncRNAs: Long noncoding RNAs
miRNAs: MicroRNAs
PAK1: P21-activated kinase 1
RAC1: Rac family small GTPase 1
RACE: Rapid-Amplification of cDNA ends
RNA-seq: RNA sequencing
TCGA: The Cancer Genome Atlas
siRNA: short interfering RNA
UTR: Untranslated region
