# Supplementary material for: Long noncoding RNA LCAT1 functions as a ceRNA to regulate RAC1 function by sponging miR-4715-5p in lung cancer
Source: Mol Cancer. 2019 Nov 29;18:171. doi: 10.1186/s12943-019-1107-y (PMC6883523; doi:10.1186/s12943-019-1107-y)
Supplement: Supplementary file 1 — Additional file 1: Table S1. List of antibodies and primers used in this study. [file 12943_2019_1107_MOESM1_ESM.pdf]

Table S1. List of antibodies and primers used in this study

Antibodies	Catalogue Number	Company
Wee1	13084	Cell Signaling Technology
CDK6	3136	Cell Signaling Technology
CyclinA2	4656	Cell Signaling Technology
CyclinB1	12231	Cell Signaling Technology
RAC1	66122-1-Ig	Proteintech
CyclinD1	2978	Cell Signaling Technology
Pak1	2602	Cell Signaling Technology
P-pak1	2606	Cell Signaling Technology
Actin	4970	Cell Signaling Technology

Targets	Primers	Sequence(5'→3')
LCAT1	Forward primer	CACTTGGGATGCTGTTTGCC
	Reverse primer	AACGATTCCTTCCGTCCCTG
miR-4715-5p	Forward primer	AAGTTGGCTGCAGTTAAGGTGG
	Reverse primer	GTCGTATCCAGTGCAGGGT
RAC1	Forward primer	CGATCCGAGCAGTCCTCT
	Reverse primer	GGGGCTGAGACATTTACAACA
RAC1 3UTR	Forward primer	ACAACGGTGGAGCCTTCGCACT
	Reverse primer	CCTCCTAACTGCAGAAGCTACCG
β-actin	Forward primer	GGCACCACACCTTCTACAAT
	Reverse primer	GCCTGGATAGCAACGTACAT

Genes	siRNA target sequences
LCAT1	GCTCAGTCAATGGGAGATT
	CCAGAGTTACAGCCAACTT
RAC1	AGACGGAGCTGTAGGTAAA
	CCGAATGAAGCGTTGCCAT
