# Supplementary material for: Long noncoding RNA LCAT1 functions as a ceRNA to regulate RAC1 function by sponging miR-4715-5p in lung cancer
Source: Mol Cancer. 2019 Nov 29;18:171. doi: 10.1186/s12943-019-1107-y (PMC6883523; doi:10.1186/s12943-019-1107-y)
Supplement: Supplementary file 2 — Additional file 2: Table S2. Quality control metrics of the RNA sequencing libraries. [file 12943_2019_1107_MOESM2_ESM.pdf]

Table S2. Quality control metrics of the RNA sequencing libraries

Sample ID	Yield (Gb)	# Reads	% of >= Q30 Bases (PF)	Mean Quality Score (PF)	% of mapping rate
A549-NC-1	9.6	67,013,960	98.0	38.6	93.7
A549-NC-2	6.9	47,052,538	97.4	38.3	92.8
A549-NC-3	9.7	65,588,240	97.4	38.3	91.8
A549-LCAT1-4-1	8.8	60,425,564	97.6	38.4	92.8
A549-LCAT1-4-2	8.2	55,637,218	97.4	38.4	93.6
A549-LCAT1-4-3	10.0	67,589,348	97.4	38.3	92.3
Calu1-NC-1	8.4	57,051,966	97.6	38.5	91.9
Calu1-NC-2	11.5	77,908,168	97.3	38.3	92.4
Calu1-NC-3	10.4	70,428,980	97.1	38.2	90.4
Calu1-LCAT1-4-1	9.5	64,882,604	97.8	38.5	84.1
Calu1-LCAT1-4-2	5.8	39,557,248	97.1	38.2	93.2
Calu1-LCAT1-4-3	8.5	58,380,246	97.5	38.4	89.6
HOP62-NC-1	10.0	68,790,834	97.8	38.5	92.8
HOP62-NC-2	10.4	70,828,680	97.4	38.3	93.4
HOP62-NC-3	10.4	70,705,350	97.3	38.3	91.7
HOP62-LCAT1-4-1	9.2	63,226,994	97.8	38.5	92.2
HOP62-LCAT1-4-2	8.5	57,844,656	97.4	38.3	93.1
HOP62-LCAT1-4-3	9.2	63,111,068	97.3	38.3	91.7
