# Supplementary material for: Long noncoding RNA LCAT1 functions as a ceRNA to regulate RAC1 function by sponging miR-4715-5p in lung cancer
Source: Mol Cancer. 2019 Nov 29;18:171. doi: 10.1186/s12943-019-1107-y (PMC6883523; doi:10.1186/s12943-019-1107-y)
Supplement: Supplementary file 3 — Additional file 3: Table S3. Characteristics of lung cancer patient samples used in the study. [file 12943_2019_1107_MOESM3_ESM.pdf]

Table S3. Characteristics of lung cancer patient samples used in the study

Characteristics	Number of cases
Age(years)	
< 65	10
≥65	15
Gender	
Male	16
Female	9
Tumor size	
< 3	12
≥ 3	13
Histology all types	
Adenocarcinomas	18
Squamous cell carcinomas	6
Others	1
