# Supplementary figures and images for: Long noncoding RNA LCAT1 functions as a ceRNA to regulate RAC1 function by sponging miR-4715-5p in lung cancer
Source: Mol Cancer. 2019 Nov 29;18:171. doi: 10.1186/s12943-019-1107-y (PMC6883523; doi:10.1186/s12943-019-1107-y)

**A**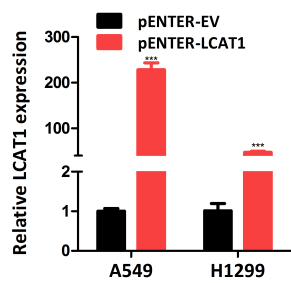**B**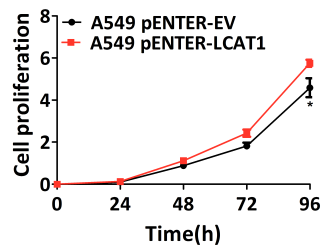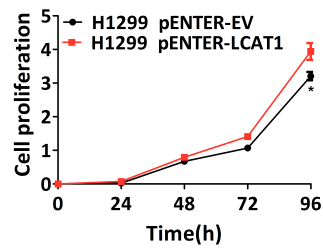**C**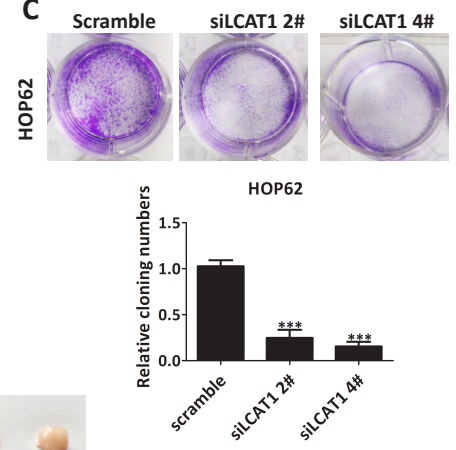**D**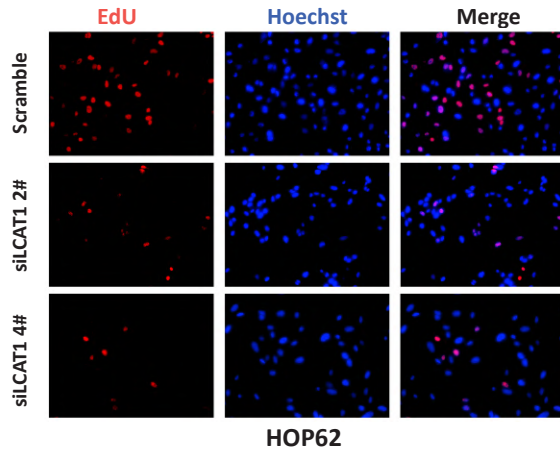**E**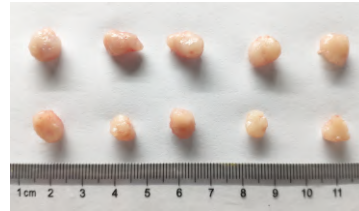**F**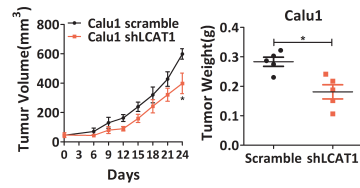

Supplement: Supplementary file 5 — Additional file 5: Figure S2. LCAT1 affects lung cancer cell proliferation. (A) Overexpression of LCAT1 in A549 and H1299 cell lines. (B) CCK8 assay was used to determine the proliferation of cells overexpressing LCAT1. (C, D) Colony formation assay and EdU assay were performed in Calu1 cells. (E, F) Tumor volume and weight of mouse xenografts subcutaneously injected with Calu1 cells with stable LCAT1 knockdown. The tumor growth curve was measured every 3 days. Nude mice were euthanized 3 weeks following treatment and the tumor nodules were collected. All in vitro experiments were performed in triplicate and one of representative results was presented. Values are expressed as mean ± SEM, *P < 0.05; ** P < 0.01; ***P < 0.001. The below is same for other figures. [file 12943_2019_1107_MOESM5_ESM.pdf]

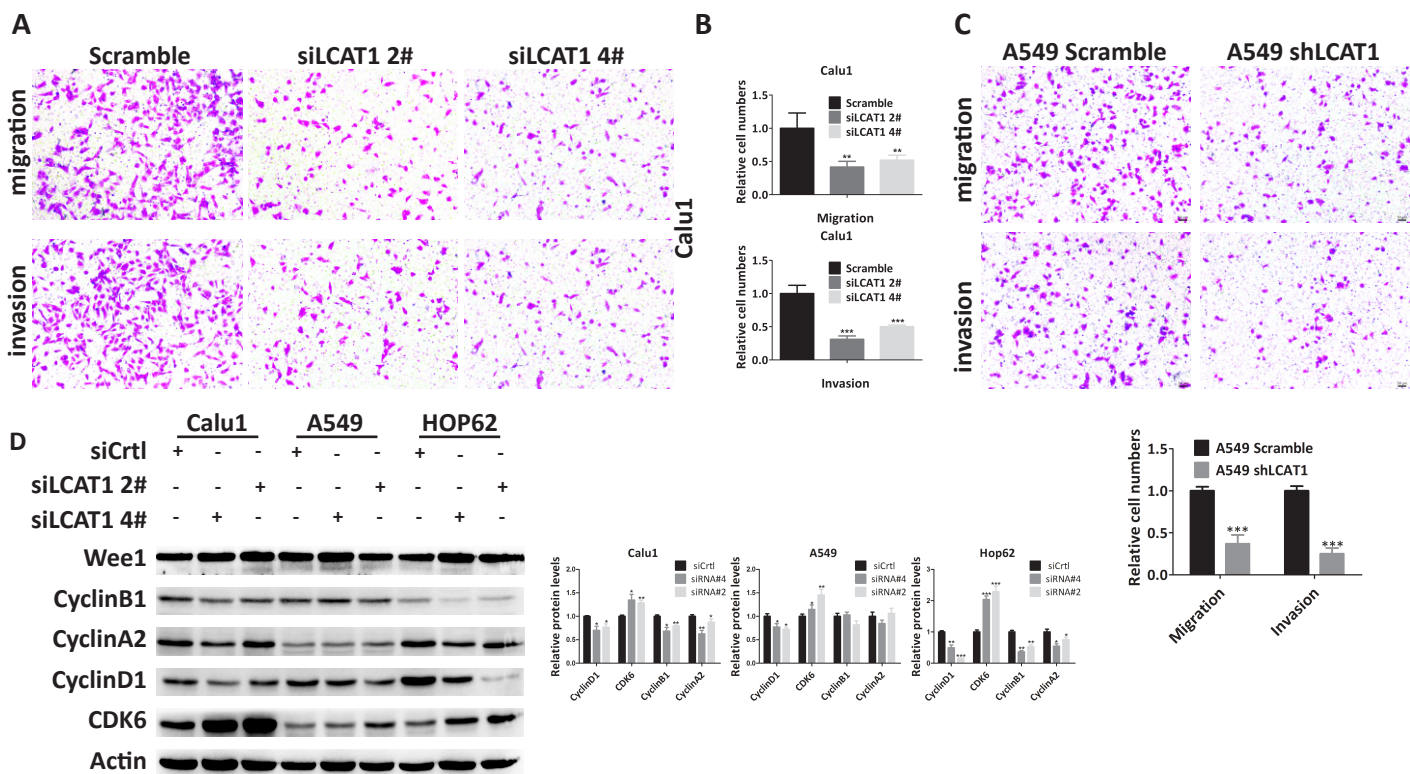

Supplement: Supplementary file 6 — Additional file 6: Figure S3. LCAT1 promotes lung cancer cell migration and invasion. (A) Representative images of transwell migration and invasion assay and (B) Number of cells between si-LCAT1 and scrambled control. (C) Transwell migration and invasion assay for A549 cells transfected with si-LCAT1. (D) Western blot analysis of cell cycle-related proteins after transfection with control siRNA, si-LCAT1 2#, or si-LCAT1 4# in the Calu1, A549 and HOP62 cells. Actin protein was used as an internal control. [file 12943_2019_1107_MOESM6_ESM.pdf]

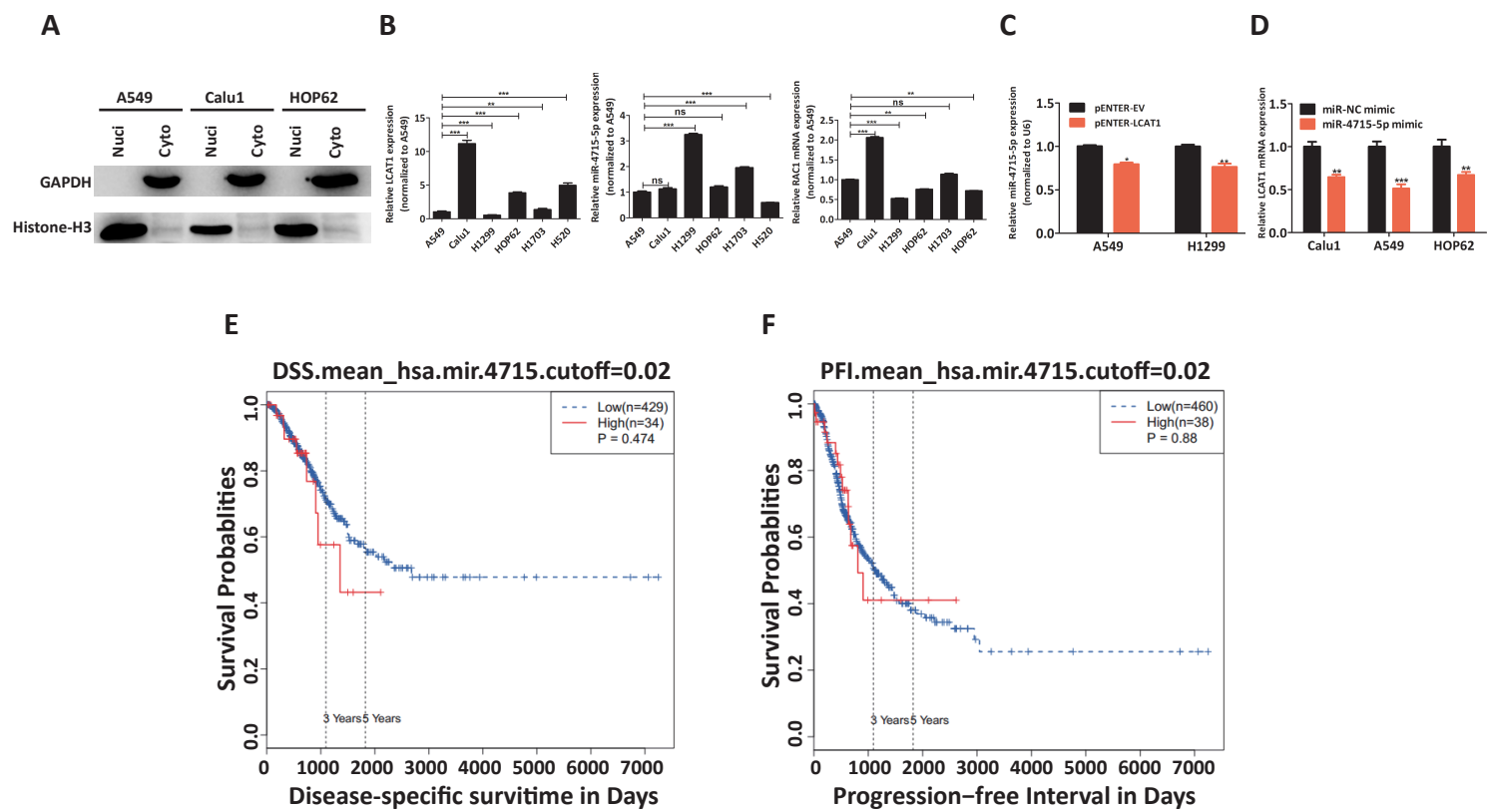

Supplement: Supplementary file 7 — Additional file 7: Figure S4. The negative correlation between LCAT1 and miR-4715-5p. (A) Western blot analysis of subcellular fraction proteins. (B) Quantification of LCAT1, miR-4715-5p, and RAC1 expression by qPCR in lung cancer cell lines. (C) Quantification of miR-4715-5p expression in the cells overexpressing LCAT1 by qRT-PCR. (D) Quantification of LCAT1 expression by qRT-PCR in cells overexpressing miR-4715-5p. (E, F) Kaplan-Meier survival analysis of overall survival and progression-free survival time in lung cancer patients based on miR-4715-5p expression. [file 12943_2019_1107_MOESM7_ESM.pdf]

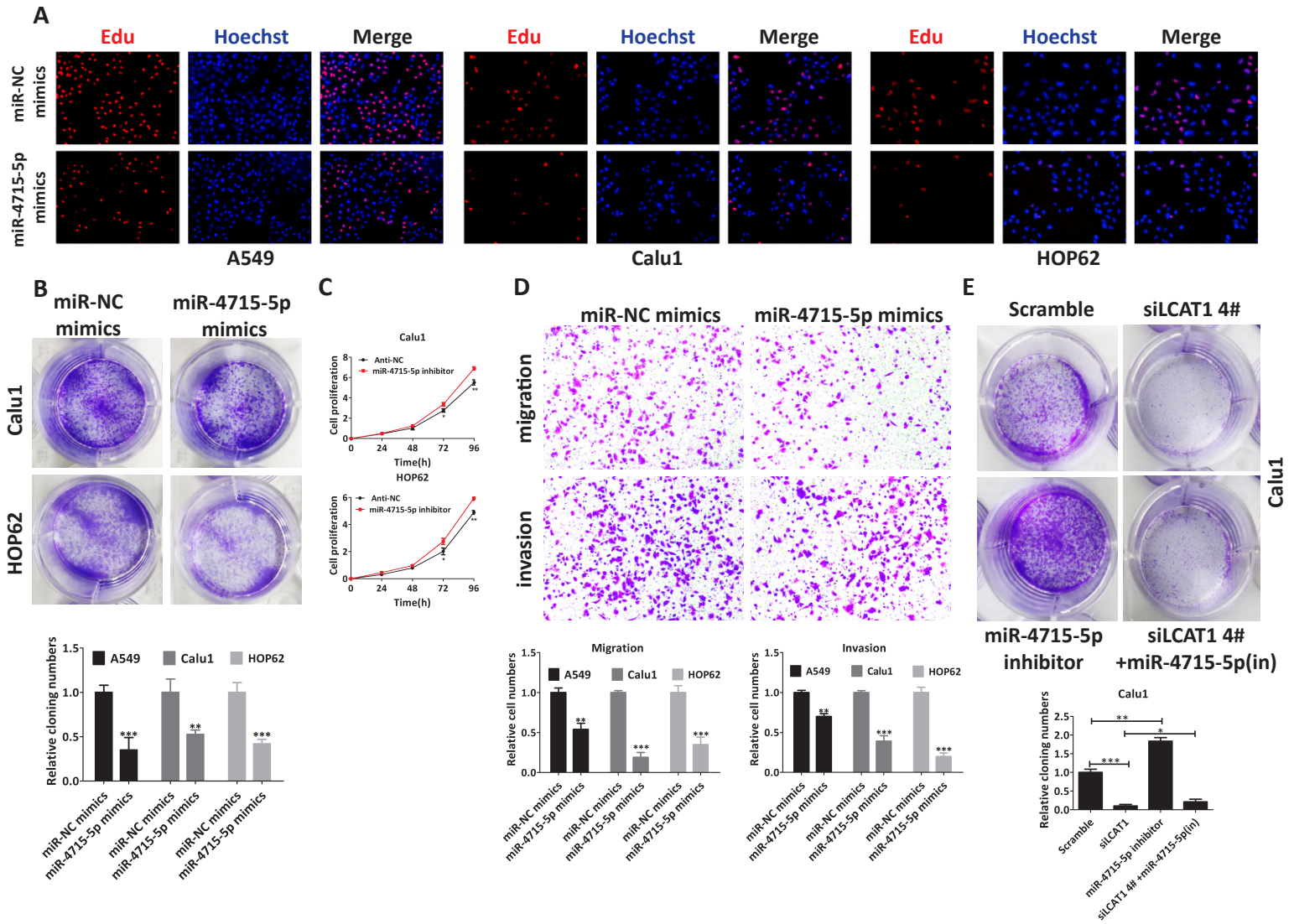

Supplement: Supplementary file 8 — Additional file 8: Figure S5. miR-4715-5p affects lung cancer cell proliferation and progression. (A) EdU assay was performed to quantify the proliferation of cells overexpressing miR-4715-5p. (B) Colony formation of cells overexpressing miR-4715-5p. (C) miR-4715-5p silencing promotes lung cancer cell proliferation. (D) Representative images of cells overexpressing miR-4715-5p from transwell migration and invasion assay. (E) Proliferation of Calu1 cells after co-transfected with control si-LCAT1–4# and miR-4715-5p inhibitor was measured using a colony formation assay. [file 12943_2019_1107_MOESM8_ESM.pdf]

**A**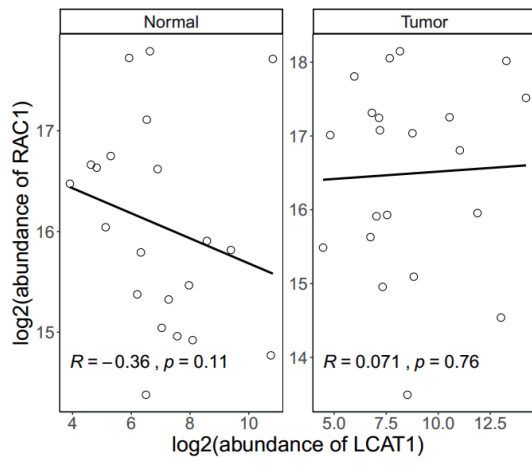**B**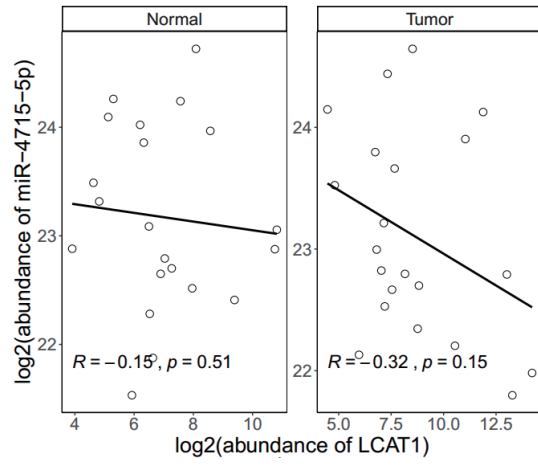**C**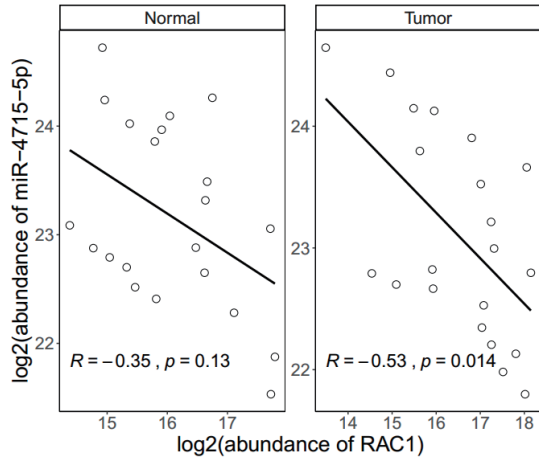**D**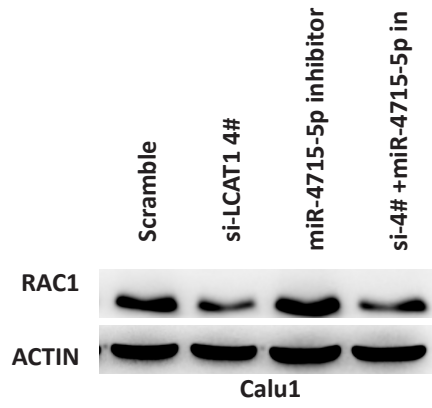

Supplement: Supplementary file 9 — Additional file 9: Figure S6. Relationship of LCAT1/miR-4715-5p/RAC1 in lung cancer tissues. (A, B, C) Pairwise correlations among LCAT1/miR-4715-5p/RAC1 in lung cancer tissues and adjacent normal tissues. The expression level of LCAT1/miR-4715-5p/RAC1 was measured by qPCR. (D) RAC1 expression in Calu1 cells transfected with si-LCAT1and miR-4715-5p inhibitor, respectively, and co-transfected with si-LCAT1and miR-4715-5p inhibitor. RAC1 expression was measured by Western blot. [file 12943_2019_1107_MOESM9_ESM.pdf]

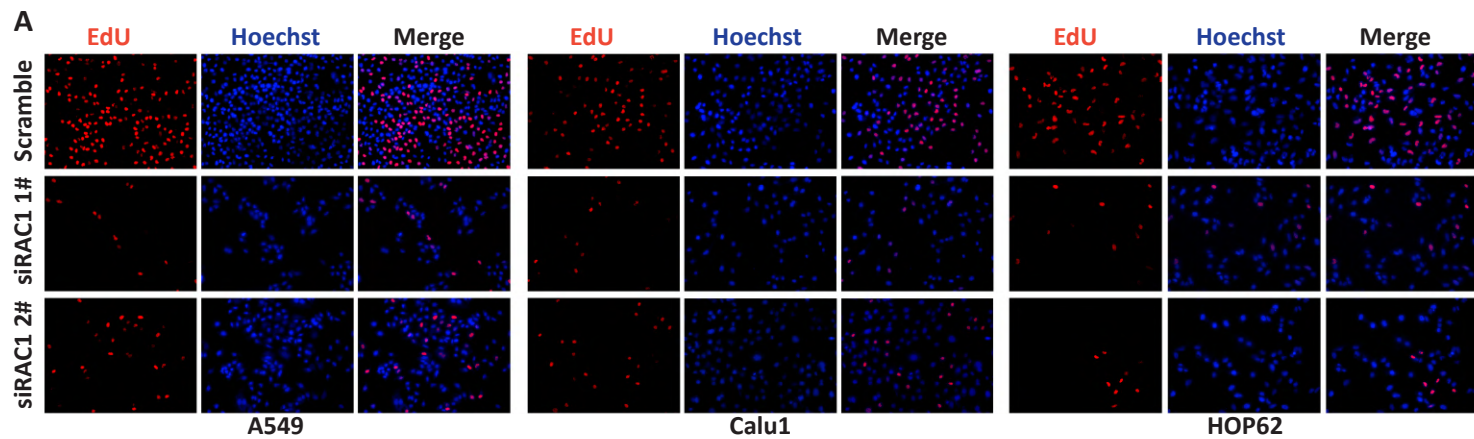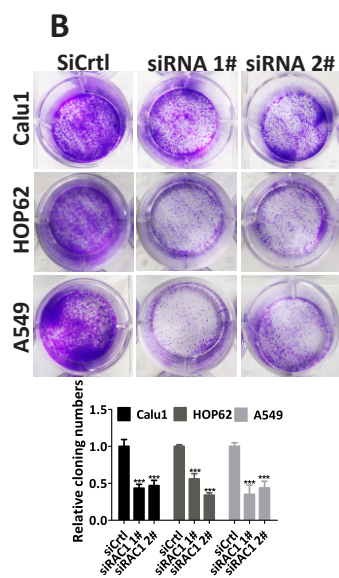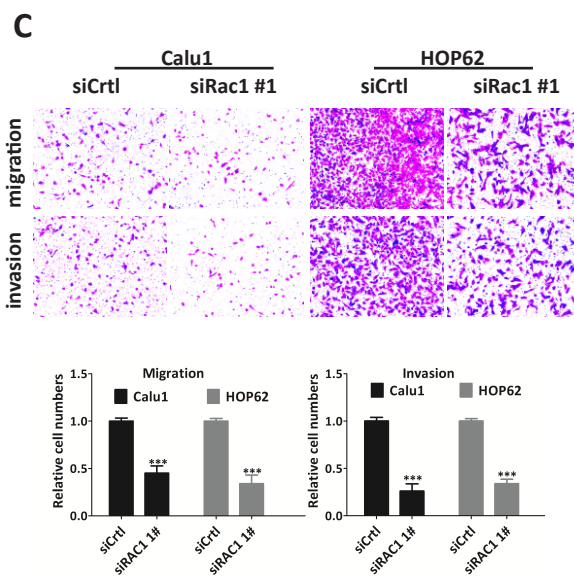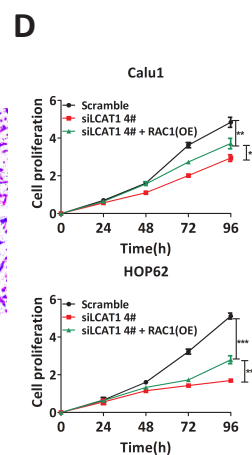

Supplement: Supplementary file 10 — Additional file 10: Figure S7. RAC1 affects lung cancer cell proliferation, invasion, and migration. (A, B) Proliferation of RAC1 knockdown cells measured by EdU and colony formation assays. (C) Migration and invasion ability of RAC1 knockdown Calu1 and HOP62 cells by transwell migration and invasion assay. (D) Proliferation of Calu1 and HOP62 cells co-transfected with siLCAT1 and pENTER-RAC1 plasmid measured by CCK-8 assay. [file 12943_2019_1107_MOESM10_ESM.pdf]
