# Supplementary material for: Long noncoding RNA LCAT1 functions as a ceRNA to regulate RAC1 function by sponging miR-4715-5p in lung cancer
Source: Mol Cancer. 2019 Nov 29;18:171. doi: 10.1186/s12943-019-1107-y (PMC6883523; doi:10.1186/s12943-019-1107-y)
Supplement: Supplementary file 4 — Additional file 4: Figure S1. RACE analysis and sequence of LCAT1. (A) Images of PCR products from the 5′ RACE and 3′ RACE. (B) Full-length sequence of LCAT1. [file 12943_2019_1107_MOESM4_ESM.pdf]

**A**

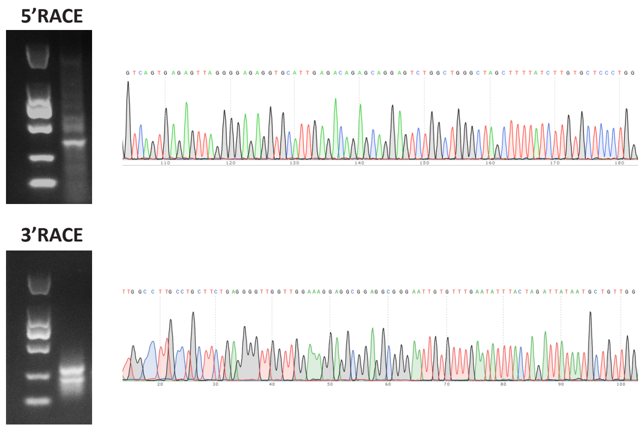


**B**

**Full length of LCAT1**

5'-GTCAGTGAGAGTTAGGGGAGAGGTGCATTGAGACAGAGCAG
GAGTCTGGCTGGGCTAGCTTTTATCTTGTGCTCCCTGGAACCCTG
ACTGGATGATTTCTGAAGTCGAGCTGGAGCCCATCTAGAGGGAA
GGTGAAACTTCTCCTCAGCATGCAGGGCCTCCTGCACCCACACTG
CCACAGCCACCGCATTACCAGCTCCTGCCAGTCTGAGGAGCAAGA
GGCTGGCATGAAGAATCAGTTCCATTCTTTCCTCCCTAAATTTAGA
AAAAGCCTGTTTTGCAAAGCTTTCTTCAGTGTTTCAAGACAAAAA
AAAAAAAATTAAGCAGTTGCACAACATGCATATCTTCTCCTGGCCT
GCAGCTCCAGGATATGCAGCTGGGAGCGAGTGTCCAGCCAACAG
CCCGTGTCTCAGGCCCTGCCAGGGGATGAATCAATTAGCAAGAGA
AGCAATGATGCAAATGCTCCGGGGTCACTTGGGATGCTGTTTGCC
TTTTTGATAGAGAAAGCACACCATAAATAACATCACACCTCAGGAA
ATCATTCCAGGAGGCCCCGATGCTGGCAGGGCTGCTGCCAGGGAC
GGAAGGAATCGTTTGCTCATTGTGTGTATTTCCCCCAGCTCAGTCAA
TGGGAGATTTGTAGTAAAATCAAAGTATTTAAAAGGAGGACAGTAT
GTGGTAACAGAGGCATGTTTAGCAATTTTTTTAGTACCAGAACCTGT
GATTTATGACCCAGAGTTACAGCCAACTTTATTACAAAAATGACTTCA
CCCATAATAACCTTATTCAGGGCCCTCTGATGAATGATGTAGTATCCAA
CAGCATTATAATCTAGTAAATATTCAAACACAATTCCCGCCTCCGCCTC
ATTTCCAACCAACCCCTCAGAAGCAGGC-3'
